# The Genetic Basis of Type 2 Diabetes in Hispanics and Latin Americans: Challenges and Opportunities

**DOI:** 10.3389/fpubh.2017.00329

**Published:** 2017-12-11

**Authors:** Josep M. Mercader, Jose C. Florez

**Affiliations:** ^1^Programs in Metabolism and Medical & Population Genetics, Broad Institute of Harvard and MIT, Cambridge, MA, United States; ^2^Diabetes Unit, Center for Genomic Medicine, Massachusetts General Hospital, Boston, MA, United States; ^3^Department of Medicine, Harvard Medical School, Boston, MA, United States

**Keywords:** genetic basis, type 2 diabetes, Hispanic, Latin Americans, heritability

## Abstract

Type 2 diabetes (T2D) affects 415 million people worldwide, and has a much higher prevalence in Hispanics (16.9%), compared to non-Hispanic whites (10.2%). Genome-wide association studies and whole-genome and whole-exome sequencing studies have discovered more than 100 genetic regions associated with modified risk for T2D. However, the identified genetic factors explain a very small fraction of the estimated heritability. Until recently, little attention has been put in studying other non European populations that suffer from a higher burden of T2D, such as Hispanics/Latinos. In the past few years, genetic studies in Hispanic populations have started to provide new insights into the genetic architecture of T2D in this ancestry group. Of note, several genetic variants that are absent or very rare in non-Hispanic populations but more common in Hispanics have shown from moderate to strong association with T2D and have provided new insights into the biology of T2D, which may be ultimately useful for developing novel therapeutic strategies applicable to all populations. Studying diverse populations can also improve the ability to find the causal variants in known T2D *loci* by a multi-ancestry fine-mapping approach, which leverages the different patterns of linkage disequilibrium between the causal and the ascertained genetic variants. In this mini-review, we summarize the main genetic findings discovered in Hispanics and discuss the limitations and challenges of performing genetic studies in these populations. Finally, we present possible next steps to make studies in Latino populations more valuable in providing a deeper understanding of T2D and anticipate their future application to the development of predictive and preventive medicine and personalized therapies.

## Introduction

Type 2 diabetes (T2D) affects more than 415 million people worldwide and is predicted to be the 7th leading cause of death in 2030 (1). T2D is particularly prevalent in Latin Americans (14.4%, twice as high as for non-Hispanic whites in the US), where it is one of the leading causes of death (2, 3). While different environmental and lifestyle risk factors in Latin America partially explain the increased prevalence of T2D, unique genetic influences also contribute (4, 5).

Genome-wide association studies (GWAS) have been able to identify more than 100 loci associated with T2D. However, until very recently, most GWAS have been performed in populations of European ancestry (6). Even in the largest trans-ancestry GWAS meta-analysis published to date, less than 40% of the samples are of non-European ancestry, and only 2% of Hispanic ancestry (7).

Genetic studies in diverse populations are essential for several reasons. First, finding a population-specific variant associated with T2D can help identify subjects at high risk for T2D in that particular population, who could be selected for lifestyle or therapeutic preventive intervention. Second, the discovery of causal genes in these populations can expand our understanding of T2D or lead to a potential therapeutic target that could be valuable even in populations where the genetic variant that prompted the discovery is not present.

During the past few years, several studies conducted in Latino populations have revealed novel associations that have improved our knowledge of the biology of T2D, and also proposed novel therapeutic targets or personalized strategies. In this review, we will describe such studies and illustrate how they might lead to potential therapeutic targets for T2D. Finally, we will suggest future research to improve the performance and interpretation of GWAS in non-European populations.

## Overview of Genetic Studies Performed in Latino Populations

The first GWAS for T2D in Hispanic populations was performed in the Mexican American population of Starr County in 2011 (8, 9). Although no novel *loci* were identified at genome-wide statistical significance (*P* < 5 × 10^−8^, selected empirically to correct for the number of independent tests among common variants in the human genome), the authors replicated several loci previously found in European populations, indicating that the majority of common genetic risk factors are transferrable to Latin American populations.

### Studies from the Slim Initiative for Genomic Medicine (SIGMA) T2D Consortium

**Figure 1:**
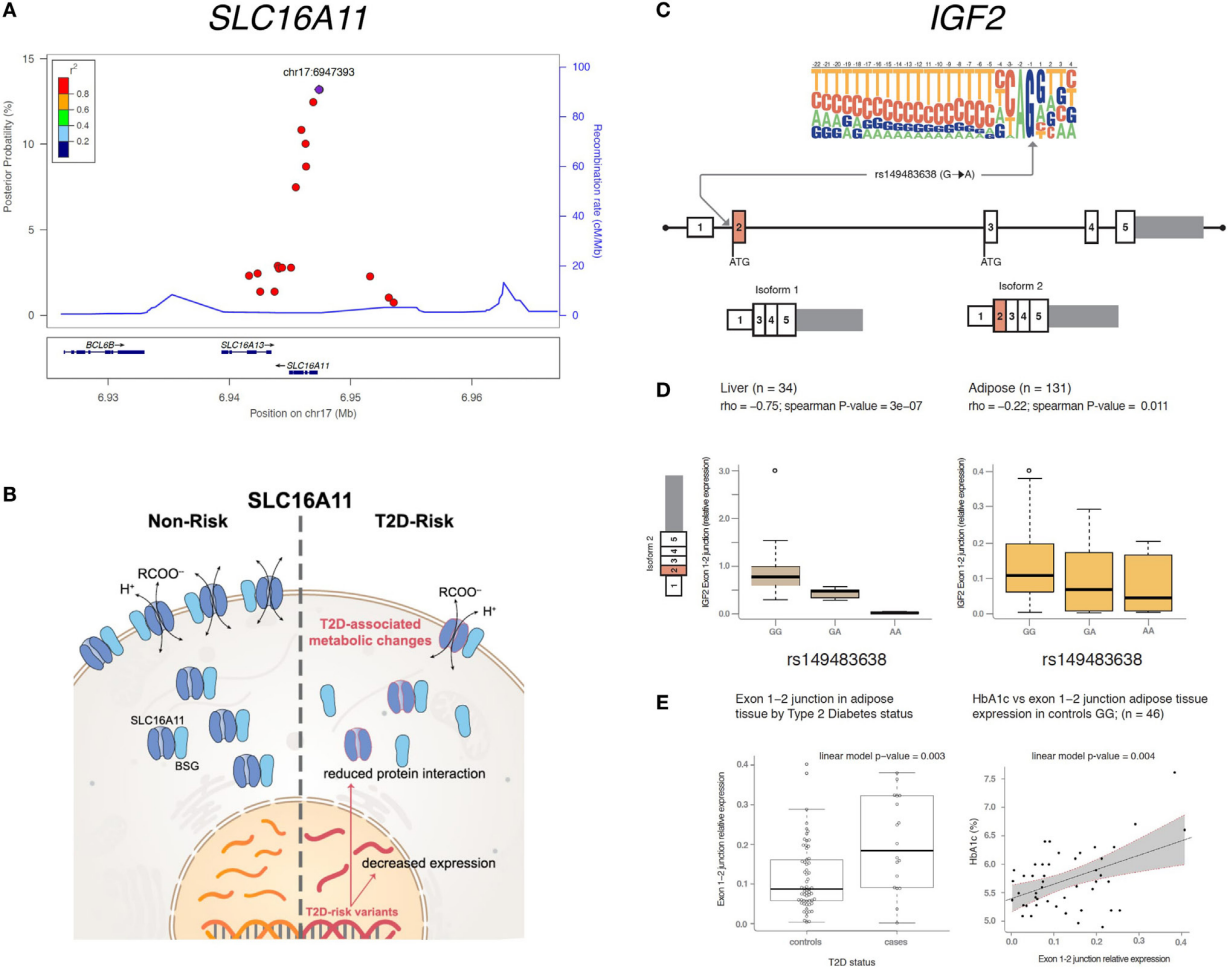
Overview of genetic associations with the potential to develop into new therapeutic strategies within SLC16A11 **(A,B)** and IGF2 **(C–E)**. **(A)** Fine mapping of the *SLC16A11* region identified in Williams et al. (4) mapped and revealed several candidate variants at or near *SLC16A11* gene. Each dot represents a variant within the 99% credible set, i.e,. the variants that have, in aggregate 99% probability of containing the causal variant. The *y*-axis represents the posterior probability of being causal, and the *x*-axis the genomic position (Hg19). The lead SNP is represented by the purple symbol. The color-coding scheme indicates the R-squared with the lead SNP, estimated based on the Mexican population. Only the SNPs that fall within the 99% credible set are plotted. **(B)** The type 2 diabetes (T2D) risk haplotype contains a *cis*-eQTL associated with lower *SLC16A11* expression in the liver. In addition, coding risk alleles in the same haplotype disrupt the interaction between SLC16A11 and basigin (BSG). Reduced SLC16A11 expression was shown to induce metabolic changes associated with T2D. **(C)** rs149483638 prevents splicing *in vitro* and *in vivo*. This variant is located at a canonical splice acceptor site, and is predicted to cause skipping of exon 2 of *IGF2* isoform 2. **(D)** The dosage of the T2D protective A allele is correlated with lower expression of *IGF2* isoform 2 (as measured by expression levels of the exon 1–2 junction) in liver and in adipose tissue. **(E)** Expression of *IGF2* isoform 2 is associated with T2D and glycated hemoglobin (HbA1c). Boxplots representing the expression of *IGF2* isoform 2 across T2D cases and controls in individuals homozygous for the G common allele. The linear model *P*-value represents the association between IGF2 isoform 2 expression, adjusted by age, body mass index, and sex. The *IGF2* isoform 2 positively correlates with higher plasma HbA1c in participants without diabetes. Figure adapted from Rusu et al. (13), Copyright 2017 by Elsevier with permission and Mercader et al. (17) American Diabetes Association and Copyright Clearance Center with permission.

As part of the SIGMA, the SIGMA T2D Consortium has shed new light on the genetic architecture of T2D in Mexicans, and resulted in several discoveries that may result in future therapeutic strategies (summarized below and in Figure 1 and Table 1).

**Table 1:** Novel genome-wide significant associations identified in Latino populations.

Target gene	Lead variant	Odds ratio (95% CI) (Latino/EA)	*P*-value (Latino/EA)	MAF (Latino)	MAF (EU)	MAF (EA)	MAF (SA)	MAF (AA)	Reference
*SLC16A11*	rs77086571	1.29 (1.20–1.38)/1.20 (1.14–1.26)^a^	5.4 × 10^−12^/7.9 × 10^−13^	0.24	0.007	0.11	–	0.0037	Williams et al. (4), Rusu et al. (13)
*HNF1A*	rs483353044	4.96 (1.75–9.92)	2.39 × 10^−9^	0.0034	0.000024	0	6 × 10^−5^	0	Estrada et al. (5)
*Insulin-like growth factor 2*	rs149483638	0.78 (0.73–0.83)	5.61 × 10^−14^	0.19	0.00008	0.008	3 × 10^−5^	0.0014	Mercader et al. (16)

*^a^ The association values in the EA population corresponds to variant rs312457, which is in high linkage disequilibrium with rs77086571 (*R*^2^ = 0.93). The association of this variant in other populations or the rest of the variants in non-Latino populations are not shown as there was not enough power to provide an accurate estimate of the effect sizes due to the low frequency in non-Latino populations*.

*MAF, minor allele frequency, taken from gnomAD (http://gnomad.broadinstitute.org/), EU, Europeans; EA, East Asian; SA, South Asians; AA, African Americans*.

#### *SLC16A11* 

In 2014, by analyzing 8,214 individuals from Mexico together with Latinos living in southern California, the first locus specific to Mexicans and Latin Americans was identified (4). This novel locus spanned the solute carrier family 16 member 11 (*SLC16A11*) and solute carrier family 16 member 11 (*SLC16A13*) genes and represented one of the strongest signals identified to date [odds ratio (OR) = 1.29, 95% CI 1.20–1.38, *P* = 5.4 × 10^−12^], in a haplotype present at ~30% frequency in Mexico, ~10% in East Asian, and rare in Europeans and African Americans. Simultaneously, a genome-wide significant signal was identified in East Asians near *SLC16A13*, in a variant which is in tight linkage disequilibrium with the top variant identified in Mexicans (*r*^2^ = 0.97), and, therefore, likely representing and replicating the same signal (10). Recent studies also suggest that this same locus is also associated with childhood T2D in Mexicans (11), it interacts with body-mass index in Native Americans, showing a stronger association in lean individuals (12).

Further fine-mapping and functional studies revealed that variants in this haplotype reduce the function of SLC16A11 by two independent mechanisms, both decreasing its expression in the liver and disrupting its interaction with basigin, a chaperone that mediates the transport of SLC16A11 to the plasma membrane. The authors also investigated the role of the SLC16A11 protein, and categorized this previously uncharacterized transporter as a proton-coupled monocarboxylate transporter. Disruption of expression of SLC16A11 induces changes in fatty acid and lipid metabolism that are associated with T2D (13). These findings suggest that increasing SLC16A11 function in the appropriate target tissue could be beneficial to prevent or treat T2D. Future work will be needed to identify the specific variants that mediate these effects and to understand how the reduction of SLC16A11 activity results in increased risk of T2D.

#### HNF1 Homeobox A Gene

The analysis of whole-exome sequences in ~3,700 individuals in the same Latino population resulted in the identification of a novel non-synonymous and population-specific variant in the hepatic nuclear factor 1 (HNF1) homeobox A gene (*HNF1A*) that was strongly associated with T2D (rs483353044, encoding p.E508K, OR = 4.96, 95% CI 2.83–10.61; *P* = 4.4 × 10^−7^), conferring one of the highest effect sizes identified at that time (5). This variant was present in 0.36% of individuals without T2D but in 2.1% of participants with the disease; because of its low frequency in a single ethnic group, it could have only been identified through sequencing approaches. This variant causes an amino acid change from glutamate to lysine in the *HNF1A* gene, one of the genes which when mutated causes maturity onset diabetes of the young (MODY). Functional exploration of transcriptional activity suggests that this variant confers an intermediate molecular phenotype between MODY and common T2D, although carriers of this variant are clinically indistinguishable from non-carriers with T2D, highlighting the need to use genetic methods to identify them. Patients with MODY who carry complete loss-of-function mutations in *HNF1A* have improved sensitivity to sulfonylureas, compared to insulin and metformin treatment (14, 15). This suggests that the 2% of individuals who carry this variant in Mexico might benefit from sulfonylurea therapy over metformin once identified by genotyping, potentially avoiding the consequences of several cycles of trial-and-error medication testing. However, pharmacogenetic studies that test this hypothesis are needed, though they need to be conducted at sites where carriers of this variant reside, necessitating the deployment of new infrastructures for “recalling by genotype.”

#### Insulin-Like Growth Factor 2

SIGMA T2D participants were also genotyped with the exome chip, which is a cost-effective genotyping array designed to capture low-frequency coding variants. This analysis identified a loss-of-function variant that was associated with 20% reduced risk of T2D (rs149483638, OR = 0.78, 95% CI 0.73–0.84, *P* = 5.6 × 10^−14^) (16). This variant was present at a frequency of ~17% in the Mexican population but was rare in Europe and other populations. The T2D protective allele disrupts a canonical acceptor splice-site that results in reduced levels of a minor isoform of insulin-like growth factor 2 (*IGF2*), isoform 2. We demonstrated that this variant prevents the expression of this isoform *in vitro*, and that carriers of this variant show reduced expression of isoform 2 *IGF2* in liver and adipose tissue *ex vivo*. Interestingly, higher expression of isoform 2 of *IGF2* in adipose tissue was associated with T2D and with increased plasma glycated hemoglobin (HbA1c) in individuals without diabetes *in vivo*. These results suggest that reducing the levels of this isoform could be a therapeutic strategy for preventing or treating T2D. The identification of loss-of-function variants associated with reduced risk of disease is of particular interest, as their protective genetic effect can be potentially recapitulated by pharmacological inhibition (which is typically easier to achieve than activation). However, to assess the viability of such a strategy, it is crucial to exclude the possibility that carriers of these T2D-protective variants might be at increased risk for other conditions, so as to reduce the chance of potential adverse effects when instituting a therapy that targets this mechanism (17). To mitigate this concern, we performed a phenome-wide association study and showed that individuals lacking this isoform did not show increased risk for a number of other common diseases nor reduced fertility, suggesting that reducing the levels of this isoform does not result in major adverse effects on health or reproduction. Because loss-of-function of this isoform is associated with reduced risk of T2D and shows no evidence of increased risk for other diseases, this isoform is proposed as a potential therapeutic target for T2D. It should be noted that the approach could be extended beyond Latin Americans, as isoform 2 of *IGF2* is expressed also in non-carriers of the rs149483638 variant. In addition, this discovery also opens a new unexplored area of research around this minor alternatively spliced isoform of IGF2, as further functional studies are needed to elucidate the precise mechanism of action of *IGF2* isoform 2 and its impact on glycemic physiology.

### Studies in Other Latin American Ancestries

Contemporary Hispanic/Latino individuals may descend from diverse ancestries, each of which may be associated differently with T2D (18). The genetic complexity of Latino or Hispanic individuals often reflects various proportions of admixed genomes from three main ancestries, including indigenous American, European, and West African. For example, due to historical circumstances Puerto Ricans or Dominicans typically possess higher proportions of African ancestry, Mexicans or Bolivians typically carry higher proportions of Native American ancestry, while Chileans or Argentinians on average carry higher proportions of European ancestry. Latino admixture occurred first between Native American and Europeans around 11 generations ago, which was followed by African admixture 7 generations ago (19). On average, Latinos in the US carry 18% Native American ancestry, 65% European, and 6.2% African Ancestry. In the US, individuals who self report as Hispanic and Mexican or Central American tend to have higher Native American ancestry than the rest of the Latinos. By contrast, those individuals reported as black, Puerto Rican, or Dominican have higher ancestries of African ancestry. Finally, those that self report as white, Cuban, or South American have higher levels of European ancestry (19).

Each of these differences in ancestries, as well as the degree of admixture, needs to be carefully taken into account to avoid artifactual associations driven by confounders (population stratification). Several methods that address the particularities of Hispanic and Latino populations have been developed and shown to overcome this type of inflation (20).

A recent study reported the GWAS results for the Hispanic Health Study/Study of Latinos (HCHS/SOL) (21). The uniqueness of this study is that it analyzed six different Hispanic ethnic groups, which provides the opportunity to identify ancestry-specific alleles associated with T2D. For example, this allowed the identification of an additional African ancestry-specific independent allele at *KCNQ1*, which was further replicated in the Meta-analysis of type 2 DIabetes in African Americans (MEDIA) consortium (22). The authors of the HCHS/SOL study acknowledge that their study was underpowered to detect modest effect sizes at genome-wide significance, but demonstrated that the previously reported 80 index variants consistently showed the same direction of effect (i. e., the risk allele was the same for the majority of the variants). Larger GWAS focusing on each of the Hispanic ancestry groups should be pursued in order to identify additional ethnic specific variants associated with T2D.

### Genetic Association with Glycemic Traits

Individuals with T2D display insulin resistance and impairment of beta-cell function and insulin secretion. Testing the association between genetic variants and additional glycemic traits can be very useful to understand the mechanism by which the associated variants increase T2D risk (23). Studying glycemic traits in European populations has provided a large number of genetic variants associated with several glycemic traits (24–26) and allowed the classification of genetic variants that influence the risk for T2D by modulating different mechanisms, including insulin secretion and insulin sensitivity. More recently, the GUaRDIAN (Genetics Underlying Diabetes in Hispanics) Consortium was formed in order to characterize the genetic components of insulin sensitivity, insulin secretion, insulin clearance, and glucose effectiveness in 4,176 Mexican Americans (27). This study revealed a genome-wide significant association at *MTNR1B* (rs10830963) with acute insulin response in a *locus* that was previously reported to be associated with fasting glucose levels in Europeans (28). Further fine-mapping performed in a total of 27,206 cases and 57,574 controls of European ancestry confirmed that the association was driven by the rs10830963 variant, which was proven to disrupt a transcription factor binding site for *NEUROD1* in the islet-derived cells EndoC-βH1 (29). A limitation of this study is that genotype imputation with 1000 Genomes reference panel was not performed, which hinders the assessment of less common variants and the translation of some of the findings described above to examine their effects on glycemic traits.

## Challenges and Limitations of Genetic Studies in Latin Americans

While substantial progress has been made in the study of non-European ancestries, including the Latin American populations, several challenges persist that limit discoveries and their follow-up in non-European ethnic groups.

### Improved Reference Panels for Imputation

One major limitation is the reduced resolution of GWAS analyses compared to which can be achieved in European populations. A key step in modern GWAS is the imputation of genotypes, for which a population-specific reference panel is required (30). The ability to impute low-frequency variants is highly dependent on the number of haplotypes that carry each given variant in the reference panel. Following this notion, larger reference panels have been assembled for the European population, such as the Haplotype Reference Consortium (31), representing 64,976 haplotypes, which enables the high-quality imputation of variants with frequencies higher than 0.1%. However, the largest source for imputation available for the Latin American population is Phase 3 of the 1000 Genomes panel, which contains only 694 individuals of Latin American ancestry (32). This places the genetic studies performed in non-European populations at a disadvantage when there is a need to identify low-frequency variants associated with complex diseases. For example, while a variant such as *HNF1A* E508K (MAF~0.36%) could be properly imputed if it was present in Europeans, this variant cannot currently be imputed in Latinos due to the lack of sufficiently large Latino-specific samples in current reference panels. To address this block, the ongoing NHLBI-funded initiative, the Trans-Omics for Precision Medicine (TOPMed, https://www.nhlbi.nih.gov/research/resources/nhlbi-precision-medicine-initiative/topmed/wgs), will release high coverage whole-genome sequencing data of 62,000 participants, of whom 10% are of Hispanic ancestry. This sequence data will eventually be very valuable as a highly precise reference panel for genotype imputation in Latin Americans.

### Availability of Functional Genomic Data for Non-Europeans

The majority of genetic associations with complex diseases, including those in T2D, fall within non-coding regions: only in a few cases can a coding variant that drives the association signal for a non-coding proxy be identified and presumed to be causal to enable the design of functional experiments. Non-coding variation is less well characterized for functional impact, which makes a mechanistic interpretation challenging. Currently, the interrogation of expression quantitative trait loci (eQTLs) is a powerful tool to assess if a non-coding variant has a regulatory effect, and additionally identify the effector genes and tissue of action for a given association. The GTEx Consortium represents one of the largest multi-tissue eQTL dataset, as it consists of genotype and gene expression data for 544 individuals and 53 tissues (33). However, a very negligible fraction of Latin American individuals are represented in this dataset, preventing the analysis of population-specific associations. Likewise, large collections of pancreatic islets gene expression and genotyping data are available for the European population, but practically none of the samples are from Latino ancestry (34). Until a similar resource such as GTEx begins to cover non-European ancestries, investigators will need to collect the tissues from the population where the variant was discovered, genotype the samples, measure gene expression of the nearby genes, and test them for association with the variant. This non-trivial experimental step introduces a substantial delay in following up association results, in contrast to a simple lookup in the GTEx portal of every variant with a frequency higher than 1% in European populations.

Therefore, significant investment is needed to assemble the first eQTL datasets including Latin American samples, perhaps starting with relevant tissues for T2D such as pancreatic islets, in order to improve the interpretation of the GWAS results in these populations.

### Large-Scale Phenotypically Rich Cohorts

Once a genetic association with a disease such as T2D is discovered, it can be very useful to analyze whether this association is specific for T2D or it is also associated with other traits or diseases. This can be specifically relevant as follow-up of loss-of-function protective variants for T2D. Before considering an association as a potential therapeutic target, phenome-wide association analysis can allow discarding the possibility that the protective variant might be associated with increased risk of other diseases or impair fertility. Large-scale biobanks with genetic data, such as the UK Biobank (35, 36), the Kaiser Permanente Research Program on Genes, Environment and Health: A Genetic Epidemiology Research on Adult Health and Aging (GERA) cohort (37), or the China Kadoorie Biobank (38) can facilitate this search. However, such a resource does not exist for Latin American populations and prevents carrying out this analysis for variants that are Latino-specific.

## Conclusion and Future Perspectives

The study of genetics of T2D in Latin Americans is still in its infancy, but has already provided exciting and promising results. The sample size of the largest studies (~9,000 cases and controls) currently analyzed in Latin American populations is comparable to those published in 2008 for Europeans, but has already provided several novel findings of potential therapeutic relevance that would also be applicable beyond the population where they were discovered. This should serve as a motivation to pursue larger-scale association analyses to find additional relevant therapeutic targets and to better understand the pathophysiology of T2D. While increasing the sample size or extending the allele frequency spectrum in European populations can provide additional novel associations for T2D, the expected population impact of the novel associations will be smaller, as the associations would have been identified earlier otherwise. However, studying different populations may provide novel associations with larger effect sizes that might be more amenable to functional studies.

In parallel to focusing on increasing the sample sizes of GWAS in Latin American populations, genomic resources that enable the functional interpretation of genetic results and building infrastructures to establish large-scale biobanks with access to genetic information will enhance the success of the discovery of potential therapeutic targets in these populations.

## Author Contributions

All authors listed have made a substantial, direct, and intellectual contribution to the work and approved it for publication.

## Conflict of Interest Statement

The authors declare that the research was conducted in the absence of any commercial or financial relationships that could be construed as a potential conflict of interest.
